# Field Verification and Multi-Site Deployment of a Multi-Sensor Node for Continuous Environmental Monitoring in Commercial Beef Cattle Facilities

**DOI:** 10.3390/ani16182862

**Published:** 2026-09-11

**Authors:** Guang Yi, Xilin Wang, Songyu Jiang, Jianfeng Zhao, Tengfei He, Zhaohui Chen

**Affiliations:** 1 College of Animal Science and Technology, China Agricultural University, Beijing 100193, China; b20233040396@cau.edu.cn (G.Y.); b20243040457@cau.edu.cn (X.W.); jsy18345753075@cau.edu.cn (S.J.); 18310753001@163.com (J.Z.); 2 State Key Laboratory of Animal Nutrition and Feeding, Beijing 100193, China

**Keywords:** beef cattle, precision livestock farming, environmental monitoring, multi-sensor node, Internet of Things, field verification, on-farm deployment

## Abstract

Continuous multi-parameter monitoring is important for characterizing environmental conditions in commercial beef cattle facilities, but sensor performance and system reliability require evaluation under production conditions. This study developed a LoRa-based multi-sensor node for air temperature, relative humidity, CO_2_, NH_3_, air speed, and illuminance. Field performance was evaluated at one commercial farm by synchronous comparison with commercial instruments using 1-min paired observations and regression- and agreement-based analyses. Nine prototype nodes were subsequently deployed at three sites for 14-day monitoring periods. Temperature and relative humidity showed the strongest linear relationships with the comparison instruments (R^2^ = 0.995 and 0.994), followed by illuminance, air speed, and CO_2_ (R^2^ = 0.990, 0.863, and 0.773). NH_3_ showed a weaker relationship (R^2^ = 0.604), improving after 10-min aggregation; most application-monitoring estimates below 5 ppm were extrapolated rather than validated absolute concentrations. Overall data availability was 99.66%, and continuous monitoring captured temporal patterns in thermal conditions, air quality, local airflow, and illuminance. The evaluation focused on environmental monitoring performance rather than animal-based outcomes. These findings support parameter-specific environmental monitoring and multi-site deployment in commercial beef cattle facilities, with potential for future anomaly detection and environmental control.

## Simple Summary

Reliable environmental information is important for managing thermal conditions and air quality in commercial beef cattle facilities, but monitoring systems are useful only when their sensors provide interpretable measurements and operate continuously under field conditions. We developed a long-range wireless multi-sensor node to measure air temperature, relative humidity, carbon dioxide, ammonia, air speed, and illuminance. The prototype was evaluated against commercial comparison instruments at one farm and subsequently deployed as nine nodes across three monitoring sites. Temperature and relative humidity showed the most stable measurement performance, while carbon dioxide, air speed, and illuminance effectively captured major temporal changes. Most ammonia estimates during application monitoring were below the sensor-specified lower bound of 5 ppm and were therefore better suited to tracking relative changes than to representing validated absolute concentrations. Overall data availability reached 99.66%, and the system consistently captured temporal patterns in thermal conditions, air quality, local airflow, and lighting. These findings support parameter-specific field verification and the practical use of the system for continuous environmental monitoring, while providing a data foundation for future anomaly detection and environmental control in commercial beef cattle production.

## 1. Introduction

Environmental conditions in cattle housing and activity areas are important factors affecting beef cattle health, productive performance, and farm management [1,2,3]. Air temperature, relative humidity, air speed, CO_2_, NH_3_, and illuminance characterize different aspects of the thermal environment, air quality, and local environmental conditions experienced by animals. High temperature and humidity increase the thermal load on cattle, while air movement affects convective heat loss and the effectiveness of cooling systems [2,4]. Variations in CO_2_ and NH_3_ concentrations are closely associated with ventilation, manure decomposition, and local air quality, whereas illuminance exhibits pronounced diurnal and management-related patterns [5,6,7]. Environmental conditions in commercial beef cattle facilities can also vary substantially over time and space. Measurements obtained from limited time points or locations may therefore fail to capture diurnal variation, short-term peaks, and abnormal environmental periods [8]. Continuous multi-parameter monitoring is thus an important basis for digital characterization of the cattle environment and for subsequent environmental risk identification and control [8,9].

In recent years, Internet of Things technologies have increasingly been applied to environmental monitoring in livestock production. Sensor nodes combined with wireless communication, gateways, and cloud-based platforms have been developed for pig, rabbit, dairy cattle, and poultry facilities, enabling continuous acquisition, storage, and visualization of parameters such as temperature, relative humidity, gaseous pollutants, illuminance, and particulate matter [10,11,12,13,14]. Environmental monitoring nodes based on microcontrollers and relatively low-cost sensors have also been evaluated against industrial-grade or commercial instruments to assess sensor performance under practical conditions [10,15,16,17]. Collectively, these studies demonstrate the potential of IoT-based systems to improve the continuity of environmental data acquisition and support data-driven farm management [9]. However, livestock species and production facilities differ considerably in building structure, ventilation strategy, environmental control, manure management, and practical constraints on sensor installation [5,6,8]. Consequently, findings obtained in other livestock systems cannot be assumed to represent the measurement performance or deployment characteristics of monitoring systems in commercial beef cattle facilities.

Field-based, simultaneous evaluation of multiple environmental sensors remains relatively limited in commercial beef cattle production. Although several livestock environmental-monitoring systems have been developed and some have undergone field or co-location evaluation, simultaneous parameter-specific assessment of measurement error and agreement under commercial beef cattle conditions remains limited [15,16,17]. This issue is particularly relevant for CO_2_, NH_3_, and air speed, which can be strongly influenced by local airflow, environmental conditions, sensor placement, and sensor response characteristics [8,17,18]. Correlation or linear regression alone may therefore be insufficient to characterize differences between prototype sensors and comparison instruments [19]. Continuous co-located monitoring, combined with regression analysis, error metrics, bias assessment, and Bland–Altman analysis, can provide a more comprehensive evaluation of field measurement performance [15,19]. In addition, deployment across independent commercial sites can provide complementary evidence regarding operational data availability and the ability of the monitoring system to capture temporal environmental patterns under different production conditions.

Recent studies have progressively addressed different aspects of intelligent environmental monitoring in commercial beef cattle facilities. Research on LoRa-based monitoring architectures has demonstrated the feasibility and reliability of end-to-end wireless data transmission under limited-connectivity conditions [3], establishing a foundation for continuous data acquisition in commercial production environments. Other studies using multipoint environmental monitoring have characterized pen-scale microenvironmental variation and further explored its relationships with cattle behavioral and physiological responses [11]. Together, these studies have advanced digital monitoring applications from reliable data transmission to environmental characterization and animal-related applications. The present study further addresses another important aspect of environmental monitoring systems by systematically evaluating the parameter-specific field measurement performance and practical deployability of a multi-sensor monitoring node through synchronous comparison with commercial instruments and subsequent multi-site deployment under commercial beef cattle conditions.

Accordingly, this study developed a LoRa-based multi-sensor node for continuous environmental monitoring in commercial beef cattle facilities, integrating sensors for air temperature, relative humidity, CO_2_, NH_3_, air speed, and illuminance, together with wireless data transmission and platform-based data management. The study had three objectives: (1) to develop a multi-parameter monitoring node suitable for field deployment in commercial beef cattle production, including sensor configuration, node structure, power supply, and enclosure design; (2) to evaluate the field measurement performance of the six environmental parameters at Farm A through synchronous comparison with commercial comparison instruments using linear regression, mean absolute error, root mean square error, bias, and Bland–Altman analysis; and (3) to deploy nine prototype nodes across three independent commercial beef cattle monitoring sites, Farms A, B, and C, and evaluate data availability and the ability of the system to characterize continuous and diurnal variation in thermal conditions, air quality, local airflow, and illuminance. Because the three sites differed in monitoring season, facility characteristics, and sensor location, they were treated as independent field application cases rather than as groups for inferential between-site comparisons. Together, these analyses were intended to define the parameter-specific field applicability and practical deployability of the system as a sensing foundation for future environmental anomaly detection and management applications.

## 2. Materials and Methods

### 2.1. Study Design and Field Deployment

The study consisted of a single-site field-verification phase and a multi-site application-monitoring phase. Field verification was conducted at Farm A in July 2025, where one designated prototype monitoring node was positioned as close as practicable to commercial comparison instruments. Air temperature, relative humidity, CO_2_, NH_3_, air speed, and illuminance were monitored simultaneously and continuously to evaluate the field measurement performance of each parameter under commercial beef cattle production conditions. Details of the commercial comparison instruments and evaluation procedures are provided in Section 2.4 and Section 2.5, respectively.

**Figure 1:**
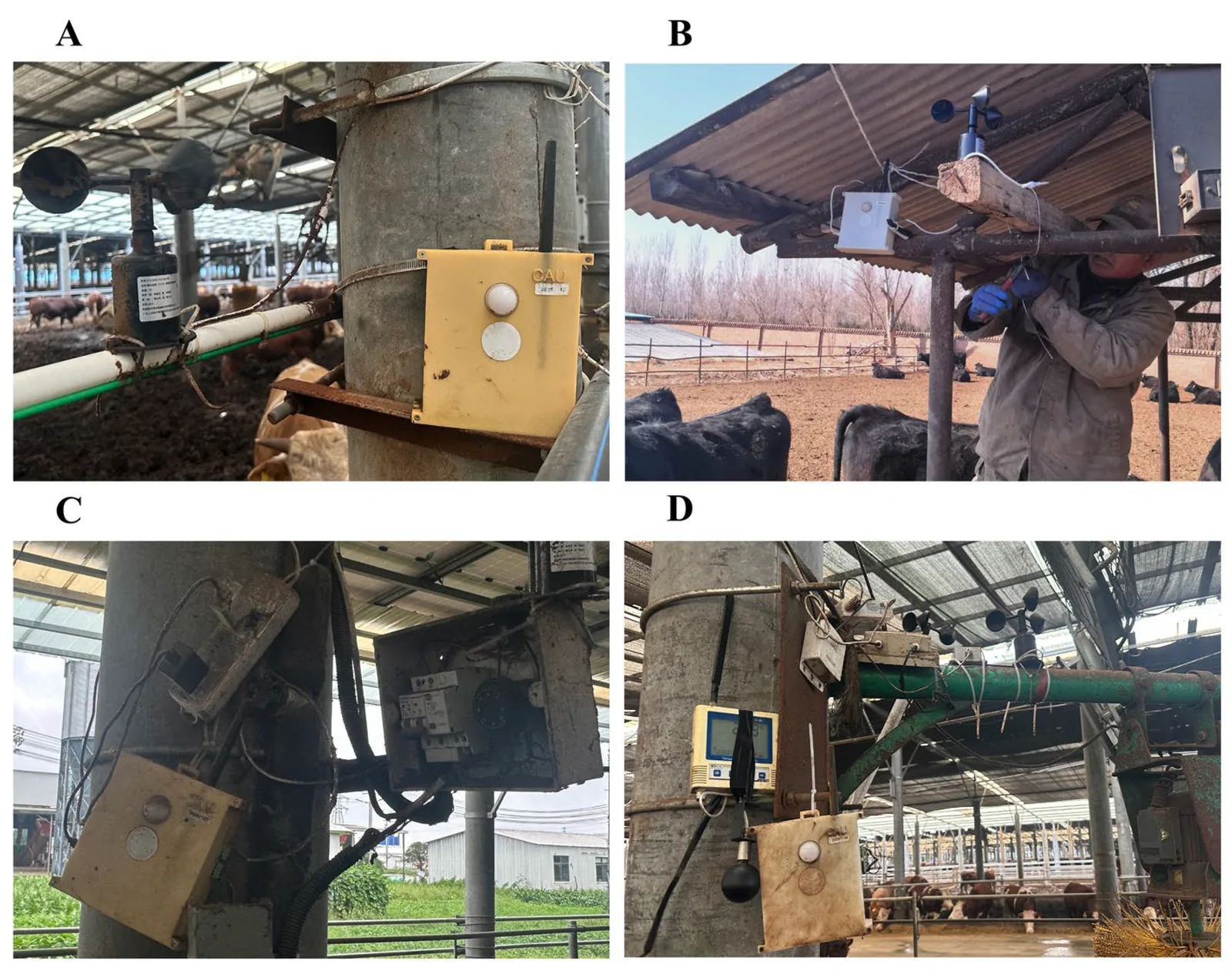
Representative field deployment of the prototype environmental monitoring system at the three commercial beef cattle monitoring sites. (**A**) Prototype-node deployment at Farm A; (**B**) deployment at Farm B; (**C**) deployment at Farm C near the drinking area; and (**D**) field-verification setup at Farm A, showing the prototype node positioned in proximity to the commercial comparison instruments. The photographs illustrate representative installation conditions and monitoring locations during field application and verification.

Multi-site application monitoring was conducted at three commercial beef cattle monitoring sites, Farms A, B, and C, using a standardized continuous 14-day monitoring period at each site. A total of nine prototype nodes were deployed, including five at Farm A and two each at Farms B and C, with all nodes operating at a base sampling interval of 1 min. During application monitoring, the sensing units at all three sites were installed at approximately 1.8 m above the barn floor or local ground surface. Node placement was determined according to facility layout, field conditions, and monitoring objectives, while direct sprinkler exposure, water flow, animal contact, and other obvious local disturbances were avoided whenever practicable. Because the three sites differed in geographic region, monitoring season, facility characteristics, and node location, they were treated as independent field application cases rather than as groups for inferential between-site comparisons. Permission to install the monitoring equipment and collect environmental data was obtained from the owner or authorized manager of each participating commercial farm before field deployment. The study role, facility type, application-monitoring period, and number of prototype nodes at each site are summarized in Table 1, and representative field deployments, including the field-verification setup at Farm A, are shown in Figure 1.

### 2.2. Development of the Multi-Sensor Environmental Monitoring Node

**Figure 2:**
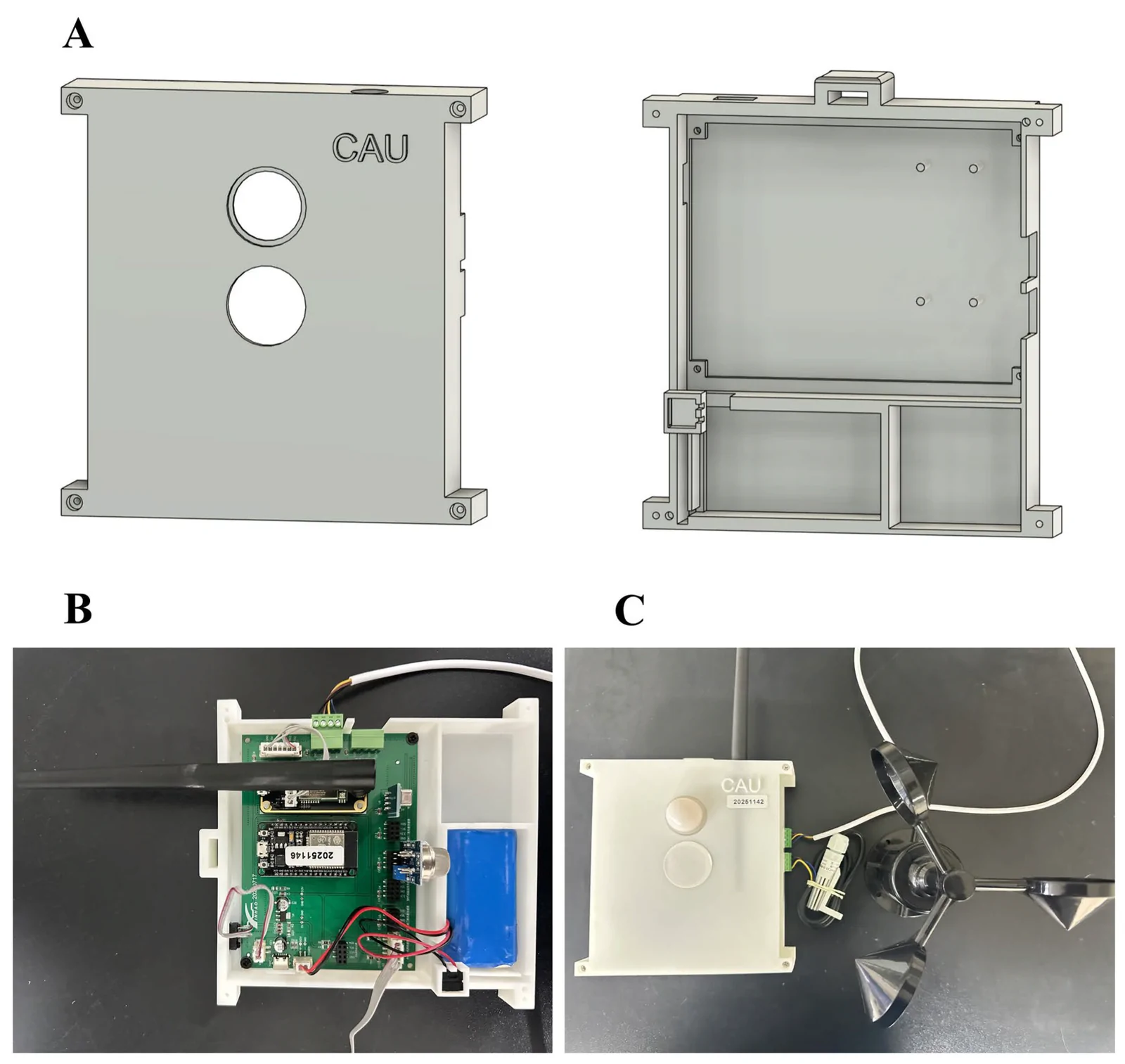
**Design and physical implementation of the multi-sensor environmental monitoring node.** (**A**) Enclosure design; (**B**) internal hardware assembly; (**C**) assembled monitoring node with the external cup-type anemometer.

The multi-sensor node integrated environmental sensing, LoRa communication, and backup power for continuous field monitoring in commercial beef cattle facilities. The node design and physical implementation are shown in Figure 2, and the integrated sensors and their main technical specifications are summarized in Table 2.

#### 2.2.1. Node Architecture and Enclosure Design

The monitoring node consisted of an ESP32-based control unit (Espressif Systems (Shanghai) Co., Ltd., Shanghai, China), sensor modules, a LoRa communication module, a power-management module, and a customized enclosure. The ESP32 was responsible for multi-sensor data acquisition, basic processing, and data-frame assembly. Digital and analog sensors were connected through the I^2^C interface and analog-to-digital converter (ADC) channels, respectively. Wireless communication was provided by an Ebyte E22-400T22S LoRa module (Chengdu Ebyte Electronic Technology Co., Ltd., Chengdu, China) operating at 433 MHz, which transmitted the acquired environmental data to the receiving gateway.

AC mains power was used as the primary power source, with a 5600 mAh 18,650 backup battery and voltage-regulation modules incorporated to maintain node operation during short-term power interruptions. Under the 1-min sampling configuration used in this study, the backup power system supported approximately 28 h of continuous node operation. The ESP32 controller, LoRa module, power-conversion components, and backup battery were secured inside the customized enclosure, with dedicated openings or ventilation windows provided for sensors requiring exposure to the surrounding environment. Considering dust, moisture, sprinkler operation, cleaning activities, and animal movement in commercial beef cattle facilities, the nodes were mounted on railings, columns, or other stable structures while minimizing direct water exposure, animal contact, and obvious local disturbances that could affect device operation or sensor measurements. The approximate hardware cost of a complete prototype monitoring node, including the sensing, processing, communication, power-supply, and enclosure components, was CNY 332; the LoRa gateway was not included in this cost.

#### 2.2.2. Sensor Configuration and Data Acquisition

The SHT35 temperature–humidity sensor and SCD41 CO_2_ sensor (both from Sensirion AG, Stäfa, Switzerland), together with the BH1750 illuminance sensor (Shenzhen Xintai Microelectronics Technology Co., Ltd., Shenzhen, China), communicated with the ESP32 through the I2C bus, whereas the MQ137 NH_3_ sensor (Shenzhen Bofengsheng Electronics Co., Ltd., Shenzhen, China) and cup-type anemometer (Jinan Zhaotaisheng Electronic Technology Co., Ltd., Jinan, China) provided analog outputs that were acquired through analog-to-digital converter (ADC) channels.

The MQ137 was selected for its straightforward integration with the ESP32-based node and its previous use for continuous NH_3_ monitoring in poultry-house environments [12]. The MQ137 is an SnO_2_-based metal-oxide semiconductor sensor with a manufacturer-specified NH_3_ detection range of 5–500 ppm. The sensor was operated using the manufacturer-recommended voltage-divider configuration. The measured output voltage (V_RL_) was converted to sensor resistance (R_s_) according to

(1) $$R_{s}=R_{L}\left(\frac{V_{C}}{V_{RL}}-1\right)$$

where R_L_ is the load resistance and V_C_ is the circuit voltage. The resulting R_s_/R_0_ ratio, where R_0_ represents the reference resistance in clean air, was converted to an estimated NH_3_ concentration using the manufacturer-provided typical sensitivity curve [20]. The curve was implemented using the log–log relationship following the approach described by Petric et al. [21]. This manufacturer-derived relationship was used for signal-to-concentration conversion rather than as an independent field calibration. Values calculated below the manufacturer-specified lower bound of the detection range (5 ppm) were retained as sensor-derived estimates but were not interpreted as validated absolute NH_3_ concentrations. The field measurement performance of the resulting NH_3_ estimates was independently evaluated against the commercial comparison instrument at Farm A.

The cup-type anemometer provided a 0–5 V analog output corresponding to an air-speed range of 0–30 m·s^−1^. The acquired signal was converted to air speed according to the sensor output relationship:

(2) u=6V

where u is air speed (m·s^−1^) and V is the analog output voltage (V). Regression relationships obtained during field verification were used only to evaluate measurement performance and were not applied as post hoc corrections to the verification data. Environmental records were generated at a base sampling interval of 1 min and included the node identifier, timestamp, air temperature, relative humidity, CO_2_, NH_3_, air speed, and illuminance.

### 2.3. Data Transmission and Platform Management

**Figure 3:**
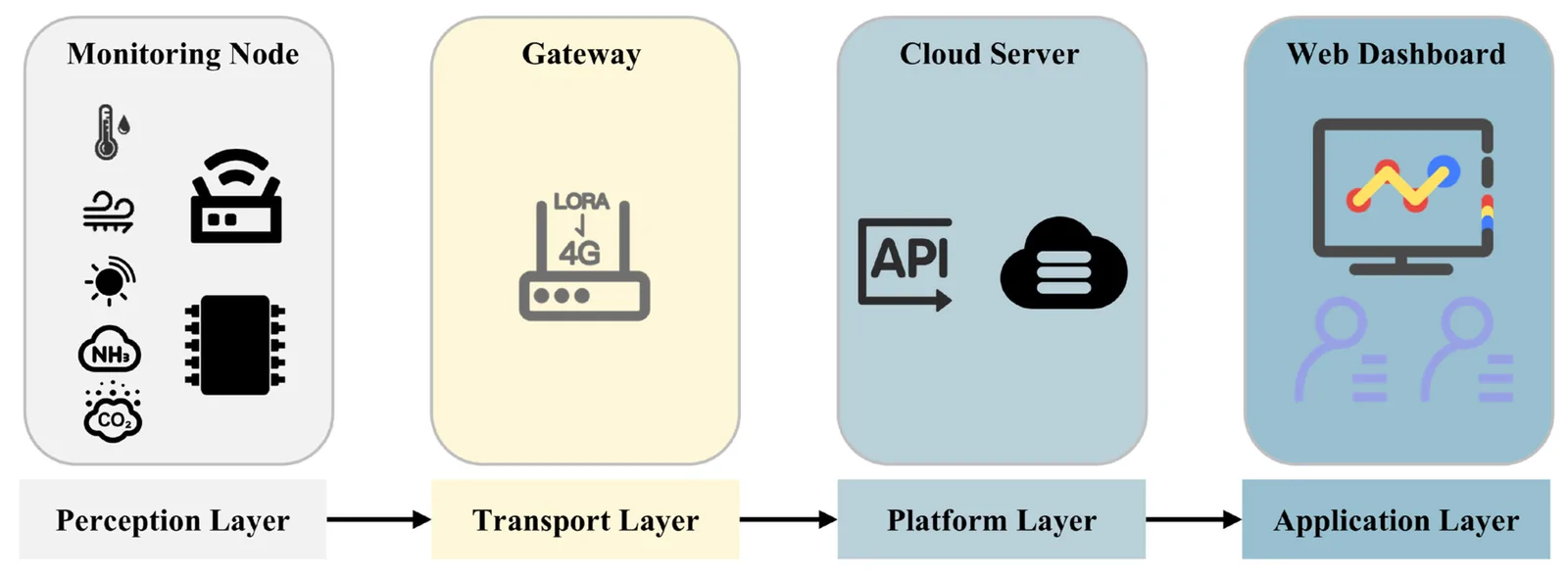
Data transmission and platform management workflow of the multi-sensor environmental monitoring system.

Environmental data collected by each prototype node were transmitted to a LoRa gateway through the E22-400T22S wireless communication module. During field deployment, LoRa communication operated at 433 MHz with a transmit power of 22 dBm, a spreading factor of SF7, and a bandwidth of 125 kHz. Environmental records were generated at 1-min intervals and transmitted from the sensor nodes to the gateway. The gateway forwarded the received records to the cloud server through a 4G cellular connection. Server-side data transmission and management were implemented through a RESTful interface and a Spring Boot-based backend (version 3.0.5) deployed on Alibaba Cloud. Received records were stored in the database and made available through the Web-based monitoring platform for subsequent data management, visualization, and analysis. The overall data transmission and platform management workflow is illustrated in Figure 3.

### 2.4. Field Verification with Commercial Comparison Instruments

To evaluate the field measurement performance of the prototype node, a standardized 10-day synchronous comparison experiment was conducted at Farm A from 7 to 16 July 2025. One designated prototype node and the commercial comparison instruments were installed at the same monitoring location, with the sensing units positioned approximately 1.8 m above the barn floor and as close to each other as practicable to minimize local spatial differences and reduce the risk of animal contact. Air temperature, relative humidity, CO_2_, and illuminance were compared with an RS-GZCO2WS-4G integrated environmental transmitter, NH_3_ with an RS-NH3 transmitter, and air speed with an RS-FSJT-4G-FV three-cup anemometer. All comparison instruments were manufactured by Shandong Renke Control Technology Co., Ltd. (Jinan, China), and their measurement ranges and manufacturer-stated accuracies are summarized in Table 3.

The prototype node recorded data at a base sampling interval of 1 min, while the commercial instruments operated continuously over the same period. Data from the two systems were matched by timestamp, and only valid paired observations were retained. If either device lacked a valid measurement at a corresponding time point, that observation was excluded from the paired analysis for that parameter; missing values were not interpolated.

Field measurement performance was evaluated using linear regression, mean absolute error (MAE), root mean square error (RMSE), mean bias, and Bland–Altman analysis [19]. For NH_3_ and air speed, 10-min aggregated data were additionally analyzed to assess the effect of temporal aggregation on regression performance and measurement agreement. Regression relationships obtained at Farm A were used only for performance evaluation and were not applied as post hoc corrections to the verification data. Detailed statistical procedures are described in Section 2.5.

### 2.5. Data Processing and Evaluation Metrics

Data exported from the server were organized into separate field-verification and multi-site application-monitoring datasets. Raw records were ordered by site identifier, node identifier, and timestamp and screened for duplicate records, timestamp anomalies, missing periods associated with device outages, and measurements outside valid or physically plausible ranges. CO_2_ observations below 400 ppm were not automatically excluded but were reviewed in relation to adjacent observations, time-series continuity, and device status; only isolated anomalous records or those associated with device abnormalities were removed. No missing values were imputed in either the field-verification or application-monitoring analyses.

For field verification, 1-min observations from the prototype node and commercial comparison instruments were matched by timestamp and retained only when both systems provided valid measurements. Missing observations were not interpolated. Parameter-specific data coverage was calculated as the percentage of final valid paired observations relative to the expected number of 1-min observations within the standardized 10-day verification window. Field measurement performance was evaluated using linear regression, the coefficient of determination (R^2^), mean absolute error (MAE), root mean square error (RMSE), mean bias, and Bland–Altman analysis. Prototype-node measurements were used as the predictor and commercial-comparison measurements as the dependent variable. MAE, RMSE, and Bias were calculated as follows:

(3) $$MAE=\frac{1}{n}\sum_{i=1}^{n}\left|P_{i}-R_{i}\right|$$

(4) $$RMSE=\sqrt{\frac{1}{n}\sum_{i=1}^{n}{\left(P_{i}-R_{i}\right)}^{2}}$$

(5) $$Bias=\frac{1}{n}\sum_{i=1}^{n}\left(P_{i}-R_{i}\right)$$

where *P*_i_ and *R*_i_ are the *i*th paired measurements from the prototype node and commercial comparison instrument, respectively, and n is the number of valid paired observations. R^2^ was used to describe the linear relationship between the two systems, whereas MAE and RMSE quantified measurement differences and bias represented the mean difference in the prototype relative to the commercial comparison instrument. The 95% confidence intervals (CIs) for MAE, RMSE, and bias were estimated using a stationary block-bootstrap procedure with 10,000 resamples to account for temporal dependence among consecutive 1-min paired observations. Parameter-specific mean block lengths were selected automatically based on the dependence structures of the paired-error, absolute-error, and squared-error series, with the largest estimated value used for the three error metrics. Percentile-based 95% CIs were defined by the 2.5th and 97.5th percentiles of the bootstrap distributions. For Bland–Altman analysis, paired differences were calculated as prototype minus commercial-comparison measurements, and the 95% limits of agreement (LoAs) were defined as the mean difference ± 1.96 standard deviations of the paired differences. To further assess magnitude-dependent dispersion, supplementary log-transformed Bland–Altman analyses were performed for relative humidity and illuminance. For positive paired observations, prototype and commercial comparison measurements were natural-log transformed, and ln(prototype/comparison) was plotted against the mean of the corresponding log-transformed measurements. Mean log ratios and 95% LoA were exponentiated to obtain prototype-to-comparison ratio bias and ratio LoA. For illuminance, the log-transformed analysis was restricted to positive paired observations because logarithmic transformation is undefined at zero. The 1-min paired observations were used for the primary analysis because they corresponded to the native monitoring interval of the system. For NH_3_ and air speed, 10-min aggregated paired observations were additionally analyzed as a secondary analysis to assess the effect of temporal aggregation on measurement agreement between the prototype and commercial comparison instruments. A 10-min aggregated value was retained only when at least eight of the ten expected 1-min paired observations (≥80%) were valid. Missing intervals were not interpolated.

Data availability during multi-site application monitoring was evaluated using all nine prototype nodes deployed at Farms A, B, and C. The expected number of records was determined from the number of deployed nodes, monitoring duration, and 1-min sampling interval. For this assessment, a record was considered valid when a timestamped node payload was successfully stored on the server and passed the predefined record-level integrity and quality-control checks. Data availability was calculated as the percentage of valid server-side records relative to the expected number of records, and missing records were calculated as the difference between the two.

For descriptive statistics and characterization of temporal environmental patterns, one designated monitoring node was selected at each site. The selected node corresponded to the primary monitoring location established for continuous environmental characterization at that site. Data from this node were used to describe temporal variation at the corresponding monitoring location and were not assumed to represent the spatial environmental distribution of the entire barn or facility. Descriptive statistics were calculated from valid, non-interpolated 1-min observations. For visualization of multi-site temporal patterns, the 1-min observations were aggregated into 10-min intervals and displayed as 14-day heatmaps. To ensure adequate temporal coverage within each interval, a 10-min mean was retained only when at least eight of the ten expected 1-min observations (≥80%) were valid. Missing observations were not interpolated.

Air temperature and relative humidity were used to calculate the temperature–humidity index (THI) according to Davis et al. [22]:

(6) $$\mathrm{THI}\;=\;0.8T\;+\;(\frac{\mathrm{RH}}{100})(T-14.4)\;+\;46.4$$

where T is air temperature (°C) and RH is relative humidity (%). Air temperature and relative humidity were summarized as mean ± standard deviation (SD) and minimum–maximum range, whereas THI was summarized as mean ± SD and maximum value. CO_2_, NH_3_, air speed, and illuminance were summarized using the median, interquartile range (Q1–Q3), and 95th percentile (P95). Air-speed measurements were interpreted as local airflow conditions at the monitoring location rather than as whole-barn or whole-facility ventilation rates. All data processing, statistical analyses, and figure generation were performed in Python (version 3.12.2).

## 3. Results

### 3.1. Single-Site Field Verification Against Commercial Comparison Instruments

After quality control and timestamp matching, 14,205–14,381 valid paired observations were retained across the six environmental parameters, corresponding to data coverage of 98.65–99.87% (Table S2). Field measurement performance for each parameter, including bootstrap-derived 95% CIs for MAE, RMSE, and bias, is summarized in Table 4.

#### 3.1.1. Temperature and Relative Humidity

**Figure 4:**
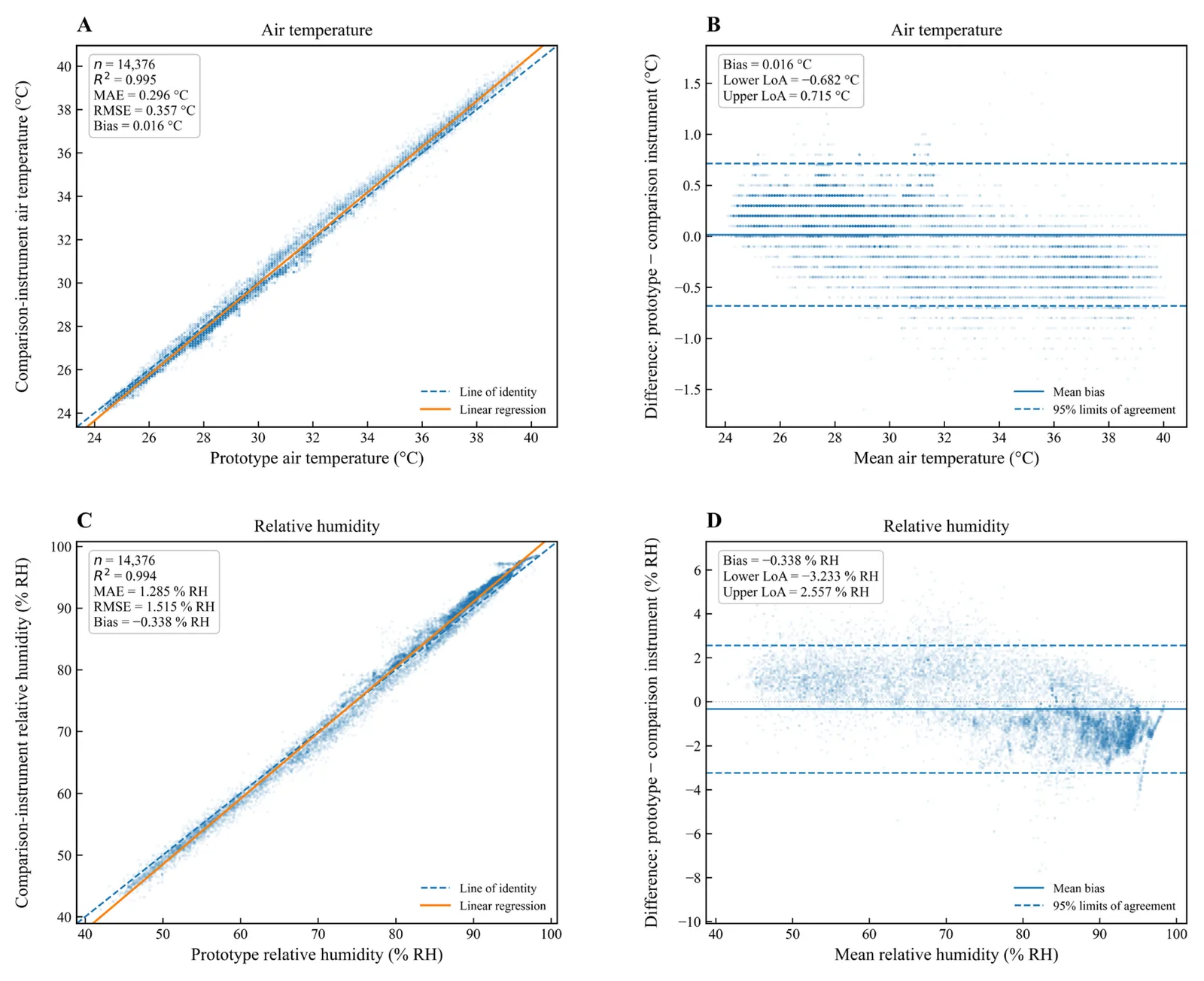
Linear regression and Bland–Altman analyses of air temperature and relative humidity during field verification. (**A**) Linear regression for air temperature; (**B**) Bland–Altman analysis for air temperature; (**C**) linear regression for relative humidity; and (**D**) Bland–Altman analysis for relative humidity. Each point represents a valid 1-min paired observation (n = 14,376 for each parameter). In panels (**A**,**C**), the dashed diagonal lines indicate the lines of identity and the solid lines indicate the fitted linear regressions. In panels (**B**,**D**), the solid horizontal lines indicate the mean biases and the dashed horizontal lines indicate the 95% limits of agreement. Bias was calculated as the prototype measurement minus the commercial-comparison measurement.

Air temperature and relative humidity showed very strong linear relationships with the commercial comparison instruments, with R^2^ values of 0.995 and 0.994, respectively (Figure 4A,C; Table 4). Mean biases were small for both parameters, at 0.016 °C for air temperature and −0.338% RH for relative humidity. Bland–Altman analysis showed that most paired differences were within the 95% limits of agreement, although relative humidity exhibited an increasingly negative bias at higher measurement levels (Figure 4B,D). In the supplementary log-transformed analysis, relative humidity showed a prototype-to-comparison ratio bias of 0.998, with ratio LoA of 0.959–1.039 (Figure S2A). Overall, both parameters showed stable field measurement performance.

#### 3.1.2. CO_2_ and NH_3_

**Figure 5:**
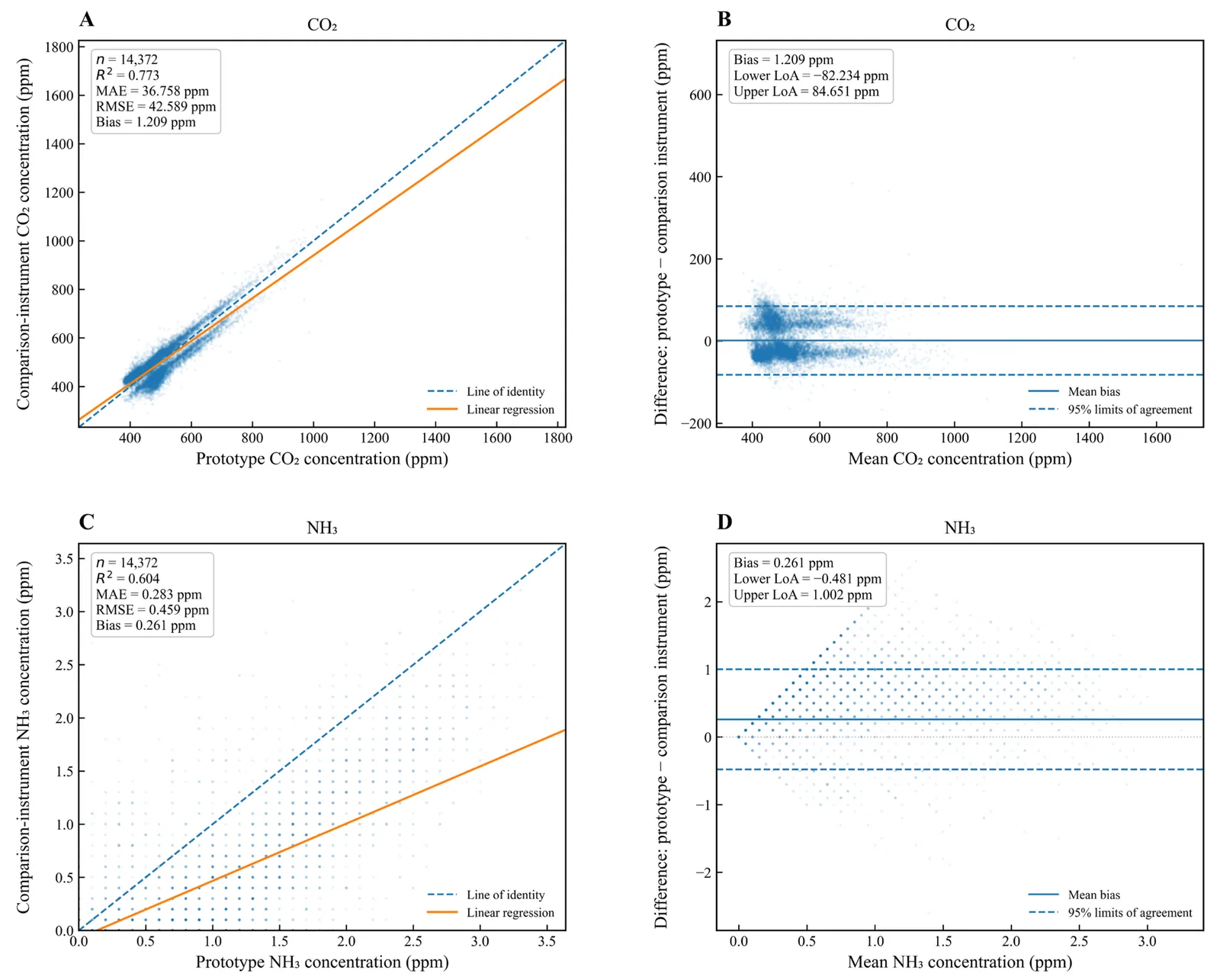
Linear regression and Bland–Altman analyses of CO_2_ and NH_3_ concentrations during field verification. (**A**) Linear regression for CO_2_; (**B**) Bland–Altman analysis for CO_2_; (**C**) linear regression for NH_3_; and (**D**) Bland–Altman analysis for NH_3_. Each point represents a valid 1-min paired observation (n = 14,372 for each parameter). In panels (**A**,**C**), the dashed diagonal lines indicate the lines of identity and the solid lines indicate the fitted linear regressions. In panels (**B**,**D**), the solid horizontal lines indicate the mean biases and the dashed horizontal lines indicate the 95% limits of agreement. Bias was calculated as the prototype measurement minus the commercial-comparison measurement.

CO_2_ showed a moderate linear relationship with the commercial comparison instrument (R^2^ = 0.773), with a mean bias of 1.209 ppm, although paired differences were more dispersed than those observed for air temperature and relative humidity (Figure 5A,B; Table 4). NH_3_ showed a weaker linear relationship (R^2^ = 0.604), with a mean bias of 0.261 ppm, and the dispersion of paired differences increased at higher measurement levels (Figure 5C,D). After aggregation to 10-min intervals, R^2^ increased to 0.755, indicating a stronger linear relationship between the two systems, although non-negligible bias and dispersion remained (Table S1; Figure S1A,B).

#### 3.1.3. Air Speed and Illuminance

**Figure 6:**
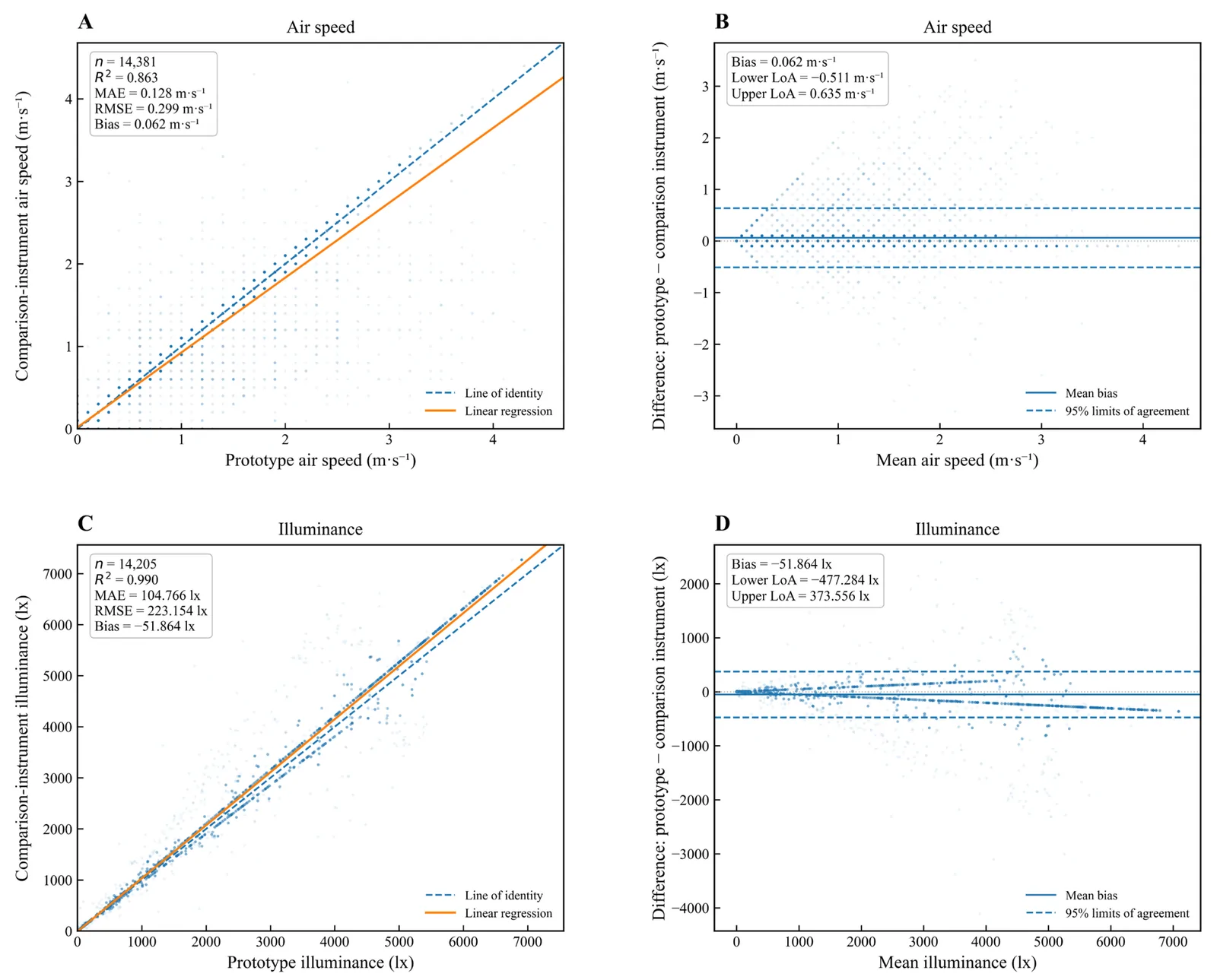
Linear regression and Bland–Altman analyses of air speed and illuminance during field verification. (**A**) Linear regression for air speed; (**B**) Bland–Altman analysis for air speed; (**C**) linear regression for illuminance; and (**D**) Bland–Altman analysis for illuminance. Each point represents a valid 1-min paired observation (n = 14,381 for air speed and n = 14,205 for illuminance). In panels (**A**,**C**), the dashed diagonal lines indicate the lines of identity and the solid lines indicate the fitted linear regressions. In panels (**B**,**D**), the solid horizontal lines indicate the mean biases and the dashed horizontal lines indicate the 95% limits of agreement. Bias was calculated as the prototype measurement minus the commercial-comparison measurement.

Air speed showed a strong linear relationship with the commercial comparison instrument at the 1-min scale (R^2^ = 0.863), with a mean bias of 0.062 m·s^−1^ (Figure 6A,B; Table 4). After aggregation to 10-min intervals, R^2^ increased to 0.977 and short-term dispersion between paired measurements was reduced (Table S1; Figure S1C,D).

Illuminance showed a very strong linear relationship with the commercial comparison instrument (R^2^ = 0.990), with a mean bias of −51.864 lx (Figure 6C,D; Table 4). Bland–Altman analysis indicated greater dispersion of paired differences at higher illuminance levels. In the supplementary log-transformed Bland–Altman analysis, 11,211 positive paired observations were retained, yielding a prototype-to-comparison ratio bias of 0.988 and ratio LoA of 0.674–1.449; magnitude-dependent dispersion was reduced but remained evident (Figure S2B).

### 3.2. Data Availability During Multi-Site Monitoring

All nine prototype nodes deployed at Farms A, B, and C were included in the data-availability assessment. Across the standardized 14-day monitoring periods, 181,440 records were expected and 180,827 valid records were retained after quality control, corresponding to an overall data availability of 99.66%. Site-specific availability exceeded 99.5% at all three monitoring sites and ranged from 99.58% to 99.85% (Table 5).

### 3.3. Continuous and Diurnal Environmental Patterns at Three Independent Monitoring Sites

**Figure 7:**
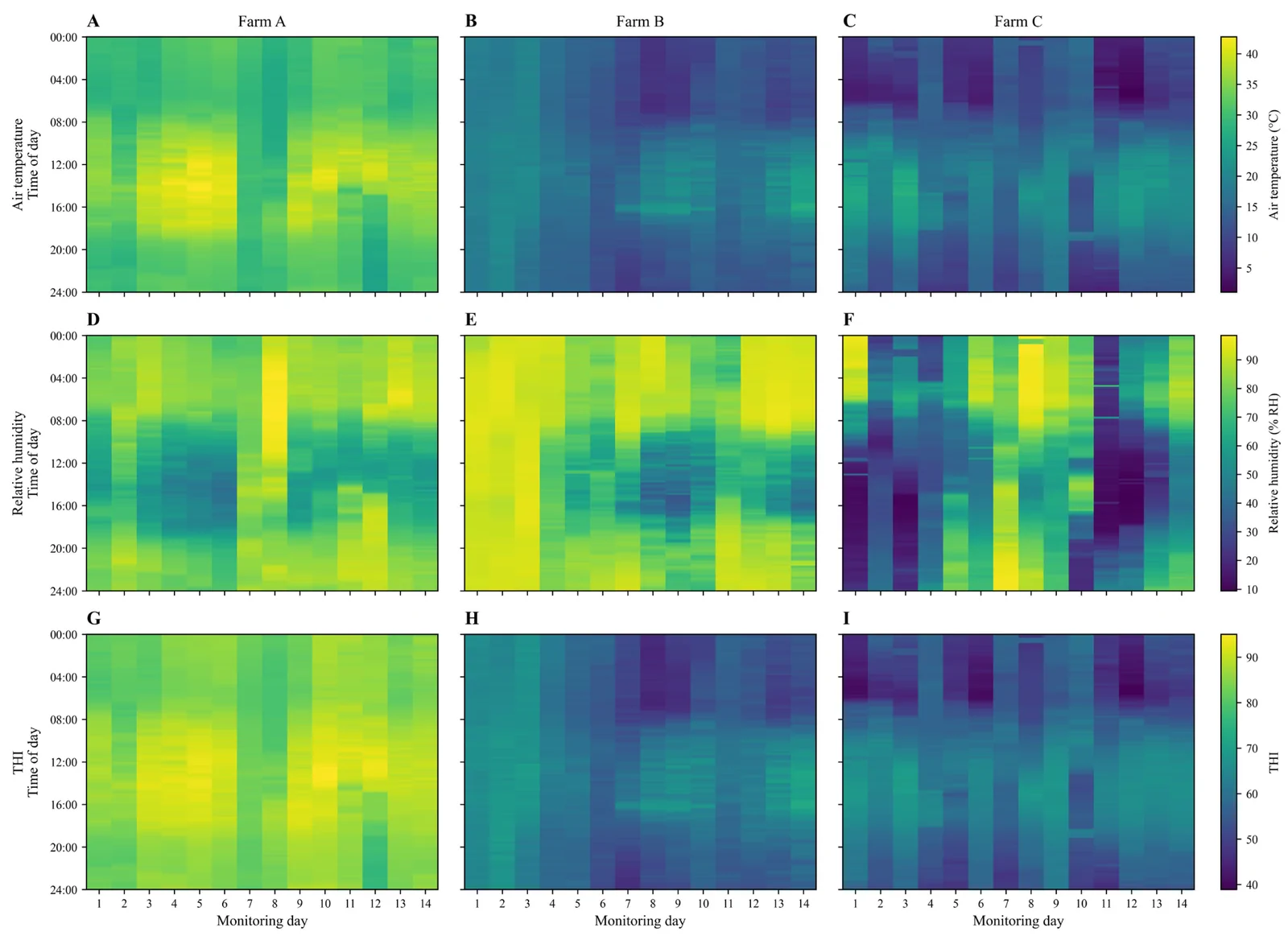
Temporal patterns of thermal-environment parameters at the three independent commercial beef cattle monitoring sites. Heatmaps show 10-min mean air temperature, relative humidity, and temperature–humidity index (THI) during the standardized 14-day monitoring periods at Farm A (**A**,**D**,**G**), Farm B (**B**,**E**,**H**), and Farm C (**C**,**F**,**I**). Ten-minute means were retained when ≥80% of the expected 1-min observations were valid; missing intervals were not interpolated. The horizontal axes represent monitoring day, and the vertical axes represent time of day. For each parameter, the same color scale was applied across the three sites. The three sites were monitored during different seasons and are presented as independent field-monitoring cases rather than for inferential between-site comparison.

**Figure 8:**
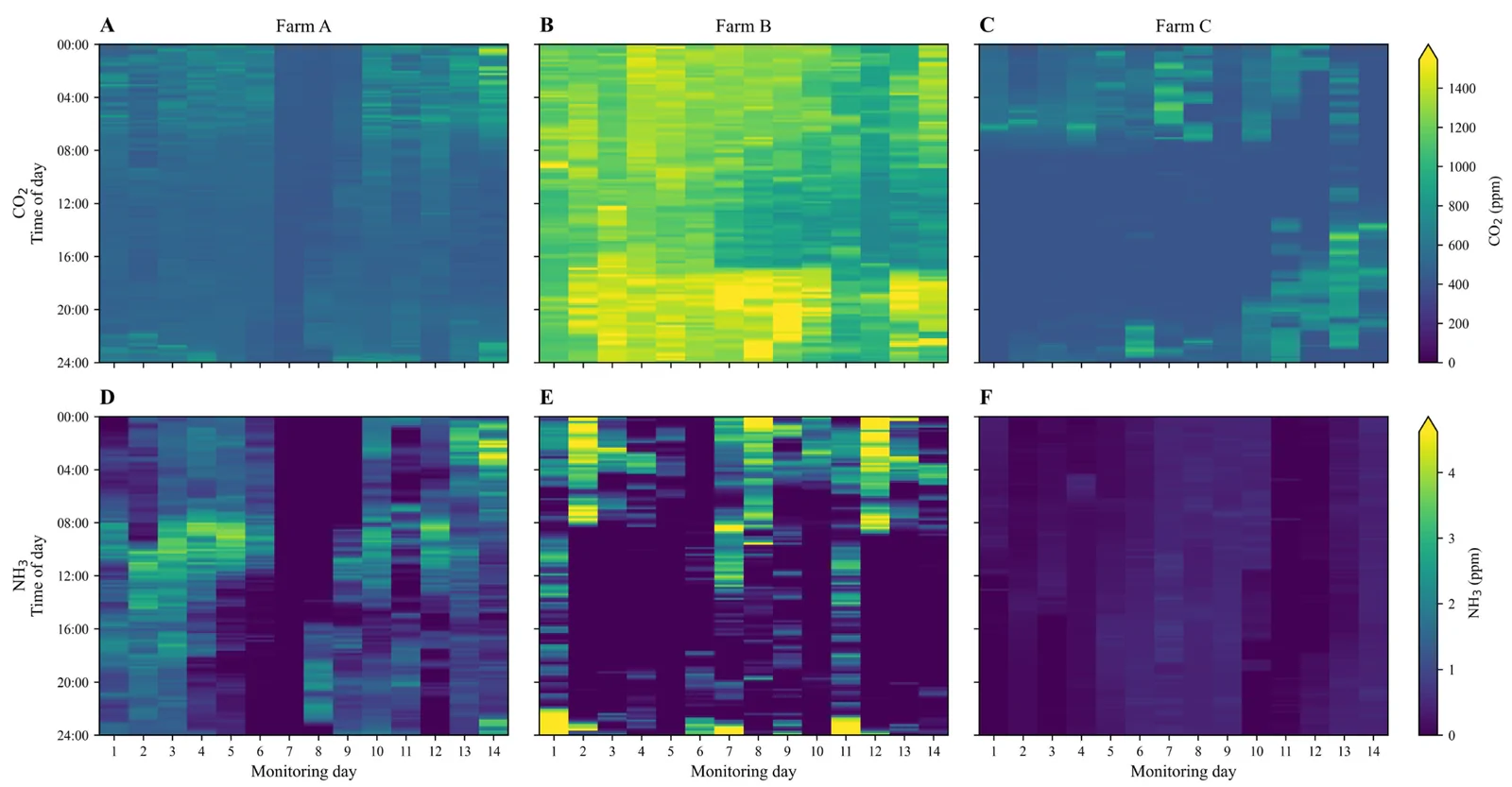
Temporal patterns of CO_2_ and NH_3_ concentrations at the three independent commercial beef cattle monitoring sites. Heatmaps show 10-min mean CO_2_ and NH_3_ concentrations during the standardized 14-day monitoring periods at Farm A (**A**,**D**), Farm B (**B**,**E**), and Farm C (**C**,**F**). The horizontal axes represent monitoring day, and the vertical axes represent time of day. For each parameter, the same color scale was applied across the three sites. Color scales ranged from zero to the 99th percentile of the pooled observations, while values above the upper limit were retained and displayed using the highest color category, as indicated by the triangular extension of each color bar. Farm C represents local conditions near the drinking area. For NH_3_, values below the manufacturer-specified lower bound of the MQ137 detection range (5 ppm) represent extrapolated sensor-derived estimates rather than validated absolute concentrations.

**Figure 9:**
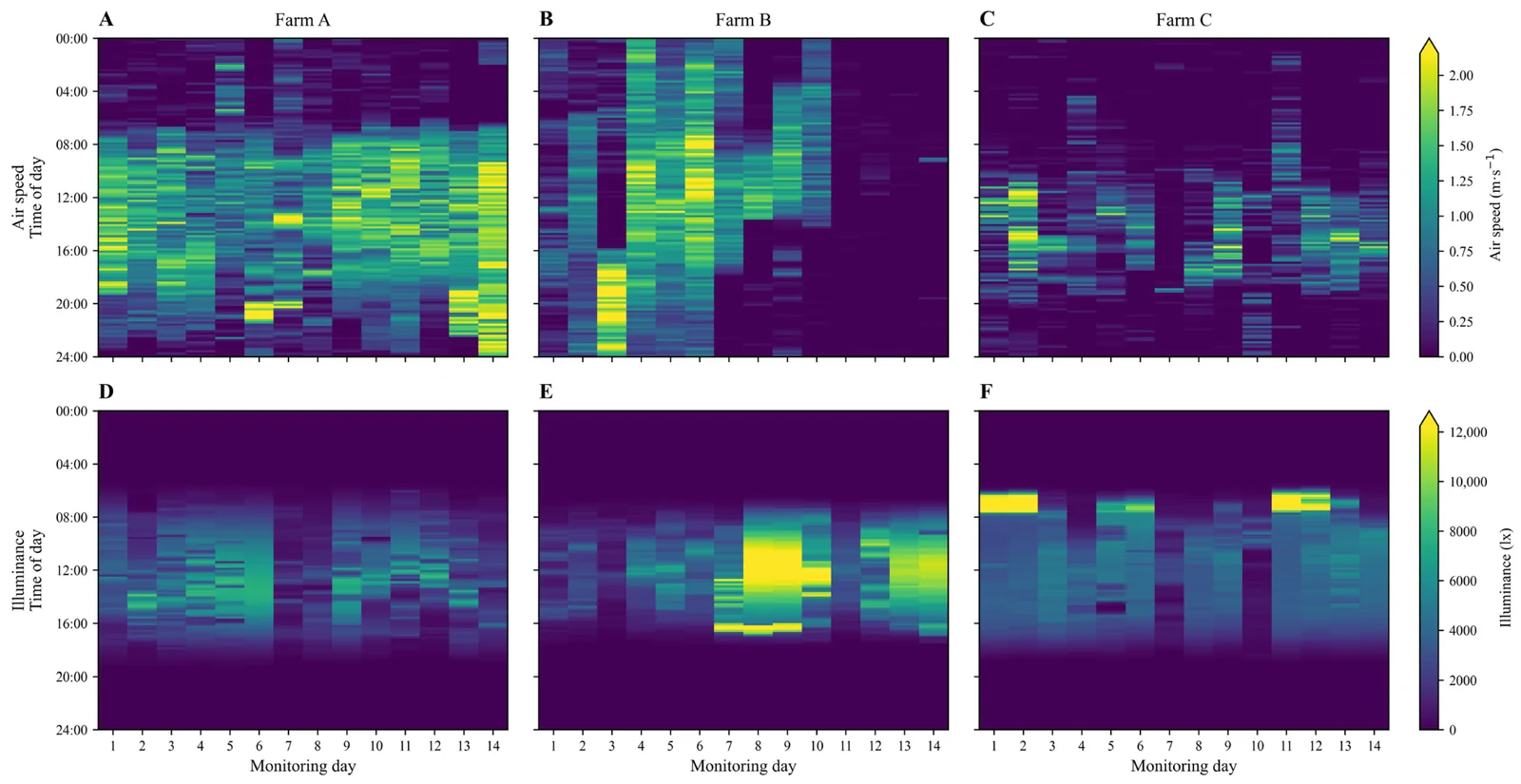
Temporal patterns of local air speed and illuminance at the three independent commercial beef cattle monitoring sites. Heatmaps show 10-min mean air speed and illuminance during the standardized 14-day monitoring periods at Farm A (**A**,**D**), Farm B (**B**,**E**), and Farm C (**C**,**F**). The horizontal axes represent monitoring day, and the vertical axes represent time of day. Air speed represents local airflow conditions at the monitoring location and should not be interpreted as a whole-barn ventilation rate. For each parameter, the same color scale was applied across the three sites. Color scales ranged from zero to the 99th percentile of the pooled observations, while values above the upper limit were retained and displayed using the highest color category, as indicated by the triangular extension of each color bar.

Descriptive statistics for the environmental parameters at the three independent monitoring sites are summarized in Table 6, and 14-day temporal patterns are shown in Figure 7, Figure 8 and Figure 9. These analyses were based on one designated monitoring node at each site, as described in Section 2.5. Because Farms A, B, and C differed in monitoring season, facility characteristics, and sensor location, they were treated as independent field application cases without inferential between-site comparisons. Data from Farm C represent local environmental conditions at the monitoring location near the drinking area rather than the spatial environment of the entire exercise yard.

#### 3.3.1. Thermal Environment

Air temperature, relative humidity, and THI showed clear diurnal variation at all three monitoring sites (Figure 7). In the Farm A summer monitoring case, mean air temperature and THI were 32.55 °C and 85.81, respectively, with higher values occurring predominantly from midday to the afternoon, while relative humidity decreased over a similar period. Farms B and C were monitored during autumn and spring, respectively, and showed lower overall air temperature and THI, while retaining clear diurnal cycles. Relative humidity at Farm C also showed pronounced day-to-day variability (Table 6).

#### 3.3.2. Air Quality

CO_2_ and NH_3_ showed distinct temporal patterns across the three monitoring cases (Figure 8). In the Farm B monitoring case, the median CO_2_ concentration was 1194 ppm, with higher concentrations occurring during the evening and nighttime on several monitoring days; the corresponding medians at Farms A and C were 513 and 414 ppm, respectively (Table 6). NH_3_ showed more intermittent temporal variation, with episodic higher values and extended low-concentration periods at Farms A and B, whereas NH_3_ estimates at the Farm C drinking-area monitoring location remained generally low and less variable.

#### 3.3.3. Temporal Patterns of Air Speed and Illuminance

Air speed and illuminance showed clear temporal variation (Figure 9). Local air movement at the Farm A monitoring location generally increased during daytime, Farm B showed more intermittent variation, and air speed near the Farm C drinking area remained low during much of the monitoring period with occasional short-term increases. Air-speed measurements represent local airflow at the sensor location and should not be interpreted as whole-barn or whole-facility ventilation rates.

All three monitoring sites showed clear diurnal cycles in illuminance, although daytime intensity and day-to-day patterns differed among monitoring cases, with short periods of high illuminance occurring on some days (Figure 9; Table 6).

## 4. Discussion

### 4.1. Field Measurement Performance and Parameter-Specific Applicability

The synchronous field comparison at Farm A demonstrated clear parameter-specific differences in the measurement performance of the multi-sensor node. The applicability of the system should therefore be defined separately for each sensing parameter rather than assuming uniform performance across all sensors. Air temperature and relative humidity showed the most stable performance and can provide a reliable basis for continuous thermal-environment monitoring and THI calculation. CO_2_ captured the major concentration changes, although potential sensor drift and environmentally induced variation should be considered during longer-term use [23,24]. Air speed and illuminance also captured the main temporal patterns; however, air speed is particularly sensitive to short-term airflow fluctuations and spatial differences between measurement locations and is therefore more appropriately interpreted as local airflow near the sensor rather than as a measure of whole-barn ventilation [25]. Previous multisensor and IoT studies in livestock facilities have similarly emphasized that measurement performance, long-term stability, and spatial representativeness differ among environmental parameters and therefore require parameter-specific field evaluation [23,24,26].

The NH_3_ measurements require greater caution. The manufacturer-specified detection range of the MQ137 is 5–500 ppm, whereas most sensor-derived NH_3_ estimates during multi-site monitoring were at the lower end of, or below, this range. Values below 5 ppm were obtained by extrapolation of the manufacturer-derived conversion relationship and should therefore not be interpreted as validated absolute concentrations. In addition, the commercial comparison instrument had limited resolution at low concentrations. Agreement under low-concentration conditions should therefore be interpreted in the context of the operating ranges and response characteristics of both sensing systems. Previous studies using MQ137 sensors have similarly emphasized the importance of sensor-specific calibration or reference-instrument verification under application conditions [27,28]. In the present study, the strength of the linear relationship increased after aggregation to 10-min intervals, although bias and dispersion remained, suggesting that the MQ137 is more suitable for identifying relative changes and short-term peaks than for high-precision absolute quantification at low NH_3_ concentrations. Collectively, these findings support a parameter-specific application strategy: temperature and relative humidity for thermal-environment and THI characterization, CO_2_ for tracking air-quality-related changes, NH_3_ for identifying relative changes and episodic peaks, air speed for local airflow characterization, and illuminance for describing the diurnal light environment.

### 4.2. Field Deployability and Interpretation of Site-Specific Environmental Patterns

Following definition of the parameter-specific applications, the multi-site deployment provided evidence of the operational performance of the system under commercial field conditions. Across nine prototype nodes at three independent monitoring sites, overall data availability reached 99.66% during the standardized monitoring periods, indicating that the data chain comprising node acquisition, LoRa transmission, and server-side storage maintained highly complete environmental time series at the deployment scale and duration evaluated in this study. Beyond data continuity, the three field cases captured site-specific temporal environmental patterns: the Farm A summer deployment showed pronounced daytime thermal load together with increased daytime local air movement, Farm B showed recurrent evening and nighttime CO_2_ elevations, and the Farm C drinking-area deployment showed generally low NH_3_ estimates and limited local air movement during much of the monitoring period. Previous multipoint monitoring studies in livestock facilities have likewise shown that continuous environmental time series can reveal temporal variation, episodic gas peaks, and spatial heterogeneity [11,29,30,31]. The value of continuous multi-parameter monitoring therefore lies not only in recording individual environmental measurements but also in characterizing how environmental conditions evolve over time.

These temporal patterns should be interpreted within the context of the individual monitoring sites. Farms A, B, and C differed in monitoring season, facility structure, geographic region, and sensor placement and were therefore treated as independent field application cases rather than as groups for inferential between-site comparison. In particular, the Farm C node was located near the drinking area of an open exercise yard and therefore represented local environmental conditions at that location. The sensing units were also installed approximately 1.8 m above the floor primarily to reduce the risk of animal contact and equipment damage. Consequently, the measurements represent conditions around the selected monitoring locations and should not be interpreted directly as animal breathing-zone exposure. Previous multipoint studies have demonstrated that CO_2_ and NH_3_ concentrations can vary spatially according to ventilation pathways and sensor location [29,30,32]. Future studies aimed at estimating animal exposure or characterizing whole-facility spatial environments should therefore incorporate additional monitoring locations and explicitly evaluate the spatial representativeness of sensor placement.

### 4.3. Practical Utility, Study Limitations, and Future Development

Taken together, the field-verification and multi-site deployment results indicate that the system is currently best positioned as a continuous multi-parameter environmental sensing tool for commercial beef cattle production. Minute-level measurements provide traceable time series for identifying periods of elevated temperature, fluctuations in gas concentrations, changes in local airflow, and diurnal lighting cycles, thereby providing a data foundation for evaluating environmental conditions and management interventions such as ventilation and cooling. In a future decision-support framework, these field-verified environmental data streams could serve as inputs for threshold- or model-based alerts and management recommendations [33,34]; however, automated decision rules or control algorithms were not evaluated in the present study. Previous livestock IoT studies have similarly demonstrated the value of continuous, remote, and multi-parameter monitoring for improving the temporal resolution of environmental information under commercial conditions [13,34,35]. Because quantitative relationships with cattle behavior, physiological responses, or productive performance were not evaluated, the current system should be interpreted as an environmental monitoring tool rather than a direct indicator of animal welfare.

Several limitations should be considered. Formal synchronous comparison of the six environmental parameters was conducted using one designated prototype unit at Farm A over a 10-day period; the remaining eight deployed prototype units were not independently co-located with a common comparison instrument, and unit-to-unit measurement variability was therefore not quantified in the present study. Application monitoring at each site covered 14 days; therefore, seasonal variation, long-term sensor drift, and maintenance requirements remain to be evaluated. The field comparison relied on commercial comparison instruments rather than traceable metrological reference devices and should consequently be interpreted as an assessment of field measurement performance rather than formal calibration. The quantitative performance of NH_3_ sensing at low concentrations and the spatial representativeness of individual monitoring locations also require further investigation. Future work should extend field verification across seasons, sites, and longer monitoring periods; improve low-concentration calibration, environmental compensation, and drift assessment for gas sensing; and use multipoint deployment to characterize spatial heterogeneity. Integration with animal-based indicators, such as respiratory rate, body-surface temperature, activity, and feeding behavior, could further improve the biological relevance of the monitoring framework and support future development of integrated risk-warning and environmental-management applications.

## 5. Conclusions

This study developed and field-verified a multi-sensor monitoring node for commercial beef cattle environments, with the evaluation focused on environmental monitoring performance rather than animal-based responses. Field comparison demonstrated parameter-specific applicability: air temperature and relative humidity showed stable performance for continuous thermal-environment monitoring and THI calculation; CO_2_, air speed, and illuminance effectively tracked major temporal changes; and NH_3_ was more suitable for identifying relative changes and short-term peaks than for high-precision absolute quantification at low concentrations. Across nine prototype nodes deployed at three independent commercial monitoring sites, overall data availability reached 99.66%, while continuous monitoring captured temporal variation in thermal conditions, air quality, local airflow, and illuminance. Overall, the system demonstrated high data availability and practical field deployability during the evaluated 14-day monitoring periods, supporting continuous multi-parameter environmental sensing and providing a data foundation for future anomaly detection and environmental control in commercial beef cattle production. Future work should extend multi-unit and long-term verification and calibration against traceable metrological reference instruments, particularly for low-concentration gas sensing and long-term sensor drift.

## Figures and Tables

**Table 1:** Study design and field deployment of the LoRa-based monitoring system at three commercial beef cattle monitoring sites.

Monitoring Site	Region	Study Role	Facility Type and Node Location	Application-Monitoring Period	Prototype Nodes
Farm A	Lixin County, Anhui Province, China	Field verification and application monitoring ^a^	Open-sided cattle barn with overhead shade	6–19 July 2026	5
Farm B	Yanqing District, Beijing, China	Application monitoring	Open-sided cattle barn with overhead shade	15–28 October 2025	2
Farm C	Lixin County, Anhui Province, China	Application monitoring	Open exercise yard; node located near the drinking area	10–23 April 2026	2

Note: ^a^ Field verification at Farm A was conducted separately from 7 to 16 July 2025 using one designated prototype node and commercial comparison instruments; the Farm A application-monitoring period shown in the table (6–19 July 2026) was a separate 14-day deployment. All application-monitoring periods lasted 14 d with a sampling interval of 1 min. Commercial comparison instruments were additional devices and were not included in the nine prototype nodes.

**Table 2:** Sensors integrated into the prototype multi-sensor environmental monitoring node.

Parameter	Sensor	Signal Type	Sensing Technology	Range	Manufacturer-Specified Performance
Air temperature	SHT35	Digital	CMOSens^®^ temperature sensing	−40–125 °C	Typical accuracy: ±0.1 °C at 20–60 °C
Relative humidity	SHT35	Digital	CMOSens^®^ capacitive humidity sensing	0–100% RH	Typical accuracy: ±1.5% RH
CO_2_	SCD41	Digital	Photoacoustic NDIR CO_2_ sensing	400–5000 ppm	Piecewise accuracy; see Note
NH_3_	MQ137	Analog, ADC	Metal oxide semiconductor gas sensing	5–500 ppm	Not specified
Air speed	Cup-type anemometer	Analog, 0–5 V/ADC	Mechanical cup rotor with electrical output	0–30 m·s^−1^	±0.3 m·s^−1^
Illuminance	BH1750	Digital	Ambient light sensor/photodiode-based sensing	1–65,535 lx	Module-specified

Note: Specifications were obtained from manufacturer documentation under the specified test conditions. SHT35 values represent typical accuracy. The manufacturer-specified SCD41 CO_2_ accuracy is ±(50 ppm + 2.5% of reading) at 400–1000 ppm, ±(50 ppm + 3% of reading) at 1001–2000 ppm, and ±(40 ppm + 5% of reading) at 2001–5000 ppm. Manufacturer-specified accuracy was not available for the MQ137. Field measurement performance was independently evaluated at Farm A.

**Table 3:** Commercial comparison instruments used for field verification at Farm A.

Parameter	Prototype Sensor	Commercial Comparison Instrument	Measurement Principle and Range	Manufacturer-Stated Accuracy	Verification Period
Air temperature	SHT35	RS-GZCO2WS-4G	Integrated temperature sensing; −40 to 80 °C	±0.5 °C at 25 °C	7–16 July 2025
Relative humidity	SHT35	RS-GZCO2WS-4G	Integrated humidity sensing; 0–100% RH	±3% RH at 60% RH and 25 °C	7–16 July 2025
CO_2_	SCD41	RS-GZCO2WS-4G	NDIR; effective range 400–5000 ppm	±(50 ppm + 3% FS) at 25 °C and 400–5000 ppm	7–16 July 2025
NH_3_	MQ137	RS-NH3, 0–500 ppm version	Non-consumptive electrochemical sensing; 0–500 ppm; resolution 1 ppm	±5% FS at 100 ppm, 25 °C and 50% RH	7–16 July 2025
Air speed	Cup-type anemometer	RS-FSJT series	Three-cup anemometer; 0–30 m·s^−1^; resolution 0.1 m·s^−1^	±(0.2 + 0.03 v) m·s^−1^ at 0–30 m·s^−1^ and 25 °C	7–16 July 2025
Illuminance	BH1750	RS-GZCO2WS-4G	Photometric sensing; 0–65,535 lx	±7% at 25 °C	7–16 July 2025

Note: All commercial comparison instruments were manufactured by Shandong Renke Control Technology Co., Ltd. (Jinan, China). FS, full scale; v, true air speed (m·s^−1^). The RS-GZCO2WS-4G integrates air temperature, relative humidity, CO_2_, and illuminance measurements.

**Table 4:** Field measurement performance of the prototype sensors relative to commercial comparison instruments based on 1-min paired observations.

Parameter	n	Slope	Intercept	R^2^	MAE (95% CI)	RMSE (95% CI)	Bias (95% CI)	Lower LoA	Upper LoA
Air temperature, °C	14,376	1.053	−1.625	0.995	0.296 (0.271 to 0.322)	0.357 (0.328 to 0.385)	0.016 (−0.057 to 0.086)	−0.682	0.715
Relative humidity, % RH	14,376	1.064	−4.680	0.994	1.285 (1.185 to 1.394)	1.515 (1.399 to 1.639)	−0.338 (−0.629 to −0.039)	−3.233	2.557
CO_2_, ppm	14,372	0.882	58.038	0.773	36.758 (32.265 to 42.018)	42.589 (37.360 to 48.327)	1.209 (−12.836 to 16.603)	−82.234	84.651
NH_3_, ppm	14,372	0.539	−0.072	0.604	0.283 (0.199 to 0.372)	0.459 (0.369 to 0.543)	0.261 (0.180 to 0.346)	−0.481	1.002
Air speed, m·s^−1^	14,381	0.907	0.018	0.863	0.128 (0.124 to 0.133)	0.299 (0.285 to 0.313)	0.062 (0.057 to 0.067)	−0.511	0.635
Illuminance, lx	14,205	1.039	−7.123	0.990	104.766 (75.147 to 135.543)	223.154 (182.104 to 260.042)	−51.864 (−78.845 to −25.391)	−477.284	373.556

Note: n represents the number of valid paired observations. Linear regression was fitted as Ycomparison = Slope × Xprototype + Intercept. MAE, RMSE, and bias are presented as point estimates with their 95% confidence intervals (CIs), estimated using a stationary block-bootstrap procedure with 10,000 resamples and parameter-specific mean block lengths. Bias was calculated as the prototype measurement minus the commercial-comparison measurement, and the 95% limits of agreement (LoAs) were defined as the mean bias ± 1.96 SD of the paired differences. MAE, mean absolute error; RMSE, root mean square error; CI, confidence interval; LoA, limit of agreement.

**Table 5:** Data availability during the standardized 14-day monitoring periods at the three commercial beef cattle monitoring sites.

Farm	Prototype Nodes	Expected Records	Valid Records	Missing Records	Availability (%)
Farm A	5	100,800	100,375	425	99.58
Farm B	2	40,320	40,194	126	99.69
Farm C	2	40,320	40,258	62	99.85
Overall	9	181,440	180,827	613	99.66

Note: Expected records were calculated from the number of deployed nodes, the 14-day monitoring duration, and the 1-min sampling interval. A record was considered valid when a timestamped node payload was successfully stored on the server and passed the predefined record-level integrity and quality-control checks. Data availability was calculated as the number of valid records divided by the expected number of records × 100.

**Table 6:** Descriptive statistics of environmental parameters at the three independent commercial beef cattle monitoring sites during the 14-day application-monitoring periods.

Parameter	Descriptive Statistic	Farm A	Farm B	Farm C
Air temperature (°C)	Mean ± SD (min–max)	32.55 ± 3.81 (24.80–43.20)	15.35 ± 3.90 (6.50–27.50)	14.22 ± 5.47 (0.95–28.46)
Relative humidity (% RH)	Mean ± SD (min–max)	76.00 ± 12.12 (39.10–98.60)	79.97 ± 14.05 (33.00–95.70)	52.75 ± 25.26 (8.66–100.00)
THI	Mean ± SD; maximum	85.81 ± 3.77; 95.79	59.26 ± 6.22; 73.69	57.04 ± 6.93; 71.58
CO_2_ (ppm)	Median (Q1–Q3); P95	513 (475–574); 749	1194 (1028–1326); 1506	414 (400–548); 814
NH_3_ (ppm)	Median (Q1–Q3); P95	1.00 (0.20–1.60); 2.80	0.00 (0.00–1.40); 3.90	0.26 (0.11–0.45); 0.56
Air speed (m·s^−1^)	Median (Q1–Q3); P95	0.60 (0.00–1.20); 2.10	0.10 (0.00–0.80); 1.90	0.00 (0.00–0.00); 1.40
Illuminance (lx)	Median (Q1–Q3); P95	324 (0–2120); 6174	8 (0–2529); 9952	312 (1–3300); 5084

Note: Air temperature and relative humidity are presented as mean ± SD with minimum–maximum ranges, and THI as mean ± SD with the maximum value. CO_2_, NH_3_, air speed, and illuminance are presented as medians with interquartile ranges (Q1–Q3) and 95th percentiles (P95). Statistics were calculated from valid, non-interpolated 1-min observations from one designated monitoring node at each site. Most NH_3_ estimates during application monitoring were below the manufacturer-specified lower bound of the MQ137 detection range (5 ppm). These values were retained to characterize relative temporal variation but represent extrapolated sensor-derived estimates rather than validated absolute concentrations.

## Data Availability

The data presented in this study are available from the corresponding author upon reasonable request.

## Supplementary Materials

The following supporting information can be downloaded at https://www.mdpi.com/article/10.3390/ani16182862/s1. Table S1: Field measurement performance of NH_3_ and air speed based on 10-min aggregated paired observations during field verification at Farm A; Table S2: Data coverage of valid paired observations used for field verification at Farm A; Figure S1: Linear regression and Bland–Altman analyses of NH_3_ and air speed based on 10-min aggregated paired observations during field verification at Farm A; Figure S2: Supplementary log-transformed Bland–Altman analyses of relative humidity and illuminance during field verification at Farm A.

## Abbreviations

The following abbreviations are used in this manuscript:

ADC	Analog-to-digital converter
I^2^C	Inter-integrated circuit
IoT	Internet of Things
LoA	Limit of agreement
LoRa	Long range
MAE	Mean absolute error
P95	95th percentile
RH	Relative humidity
RMSE	Root mean square error
SD	Standard deviation
THI	Temperature–humidity index
CI	Confidence interval
