# Supplementary material for: Field Verification and Multi-Site Deployment of a Multi-Sensor Node for Continuous Environmental Monitoring in Commercial Beef Cattle Facilities
Source: Animals (Basel). 2026 Sep 11;16(18):2862. doi: 10.3390/ani16182862 (PMC13603164; doi:10.3390/ani16182862)
Supplement: Supplementary file 1 [file animals-16-02862-s001.zip › animals-4531204_Supplementary_Materials_Proofread_Tracked.pdf]

Table S1. Field measurement performance of NH_3 and air speed based on 10-min aggregated paired observations during field verification at Farm A.

Parameter	n	Slope	Intercept	R^2	MAE	RMSE	Bias	Lower LoA	Upper LoA
NH_3, ppm	1,440	0.563	−0.082	0.755	0.265	0.406	0.261	−0.349	0.870
Air speed, $m \cdot s^{-1}$	1,440	0.967	−0.034	0.977	0.087	0.118	0.062	−0.136	0.260

Note: n represents the number of valid 10-min paired observations. Each 10-min value was calculated from the available valid paired 1-min observations within the corresponding 10-min interval and was retained only when at least eight of the ten expected 1-min paired observations (≥80%) were valid; missing 1-min observations were not interpolated. Linear regression was fitted as $Y_{comparison}$ = Slope × $X_{prototype}$ + Intercept. Bias was calculated as the prototype measurement minus the commercial-comparison measurement, and the 95% limits of agreement (LoAs) were defined as the mean bias ± 1.96 SD of the paired differences. MAE, mean absolute error; RMSE, root mean square error; LoA, limit of agreement.

Table S2. Data coverage of valid paired observations used for field verification at Farm A.

Parameter	Expected records	Final matched pairs	Time points without a valid matched pair	Coverage (%)
Air temperature	14,400	14,376	24	99.83
Relative humidity	14,400	14,376	24	99.83
CO_2	14,400	14,372	28	99.81
NH_3	14,400	14,372	28	99.81
Air speed	14,400	14,381	19	99.87
Illuminance	14,400	14,205	195	98.65

Note: Expected records were calculated for the standardized 10-day verification window from 7 July 2025 00:00 (inclusive) to 17 July 2025 00:00 (exclusive), with a 1-min sampling interval, yielding 14,400 expected observations for each parameter. Final matched pairs represent time points for which both the prototype node and the corresponding commercial comparison instrument provided valid observations after timestamp alignment. Time points without a valid matched pair include missing, invalid, or unmatched observations from either device. Missing observations were not interpolated. Coverage was calculated as the number of final matched pairs divided by the expected number of records × 100.

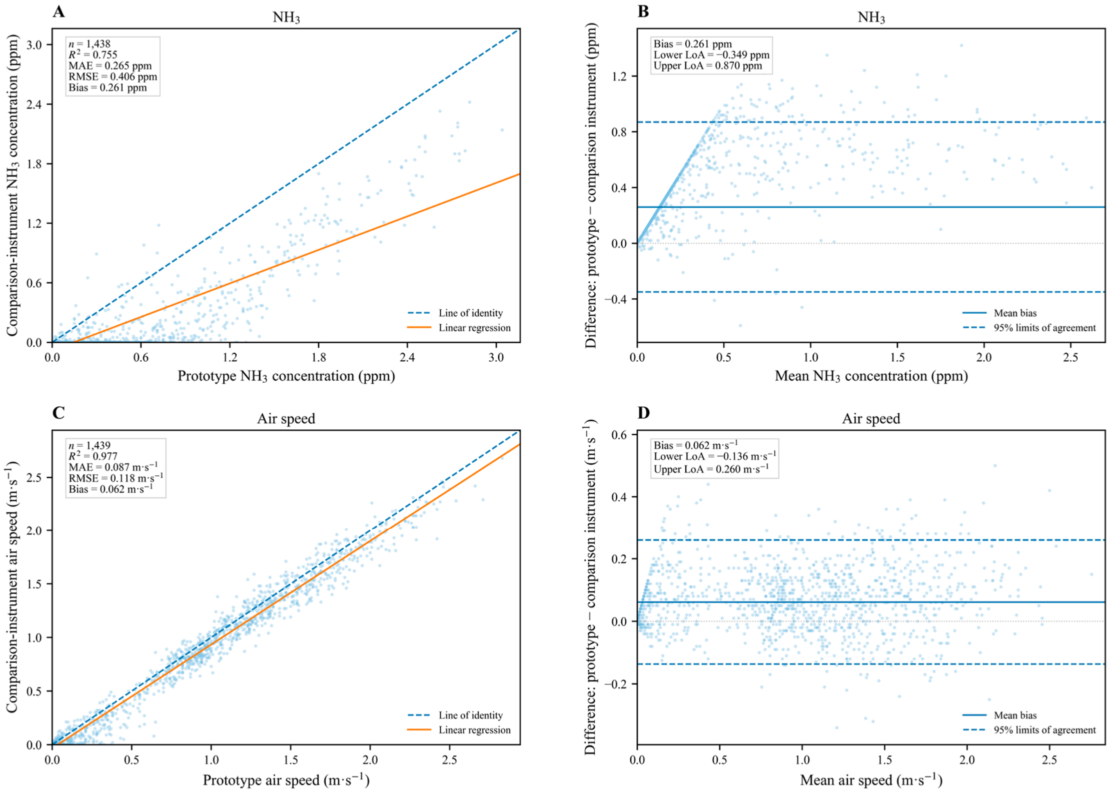

Figure S1. Linear regression and Bland–Altman analyses of NH_3 and air speed based on 10-min aggregated paired observations during field verification at Farm A. (A) Linear regression for NH_3; (B) Bland–Altman analysis for NH_3; (C) linear regression for air speed; and (D) Bland–Altman analysis for air speed. Each point represents one valid 10-min paired observation. In panels A and C, the dashed diagonal lines indicate the lines of identity and the solid lines indicate the fitted linear regressions. In panels B and D, the solid horizontal lines indicate the mean biases and the dashed horizontal lines indicate the 95% limits of agreement. Bias was calculated as the prototype measurement minus the commercial-comparison measurement.

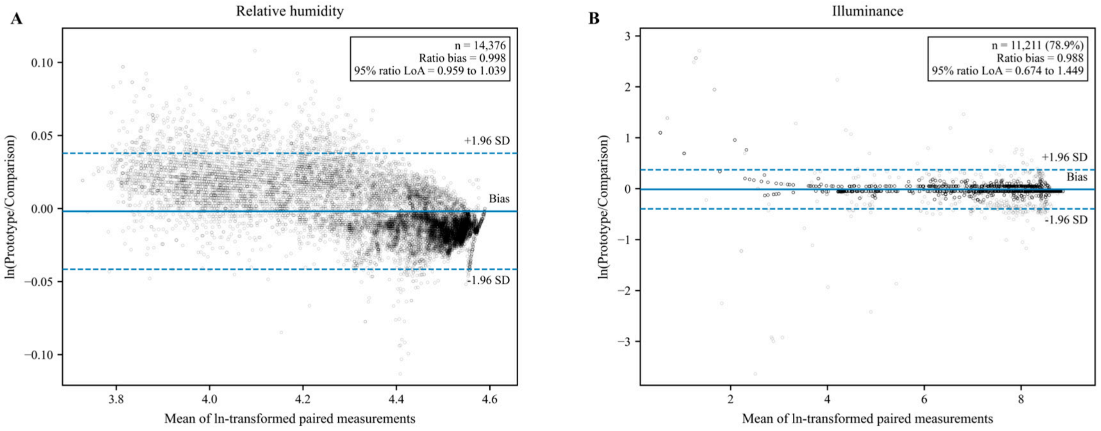

Figure S2. Supplementary log-transformed Bland–Altman analyses of relative humidity and illuminance during field verification at Farm A. (A) Relative humidity and (B) illuminance. Prototype and commercial comparison measurements were natural-log transformed, and the log ratio, ln(prototype/comparison), was plotted against the mean of the corresponding log-transformed paired measurements. Solid horizontal lines indicate the mean log ratio (bias), and dashed horizontal lines indicate the 95% limits of

agreement (LoAs). Back-transformed values are reported as prototype-to-comparison ratio bias and ratio LoA. All 14,376 paired observations were retained for relative humidity. For illuminance, 11,211 positive paired observations (78.9% of the original 14,205 pairs) were included because logarithmic transformation is undefined when either paired measurement is zero.
